# The “phantom” rash of Still's disease

**DOI:** 10.11604/pamj.2015.22.157.8144

**Published:** 2015-10-20

**Authors:** Theocharis Koufakis, Ioannis Gabranis

**Affiliations:** 1 Department of Internal Medicine, General Hospital of Larissa, Larissa, Greece

**Keywords:** Still′s disease, rash, fever

## Image in medicine

An 18-year-old, female patient presented with fever, sore throat and joint pain. Laboratory tests revealed elevated inflammation markers. During her hospitalization she presented a non-pruritic, salmon-colored rash, which was appearing with the fever in the evening hours and was vanishing during apyrexia. The rash could be seen in various parts of the body, such as the limbs, the face and the neck. The diagnosis of Adult-onset Still disease (AOSD) was established, since the patient was fulfilling the relevant criteria. AOSD is a rare, systemic inflammatory disorder of unknown etiology that typically presents as a high spiking fever accompanied by systemic symptoms. Various skin lesions have been described in patients with AOSD, both typical and atypical ones. Our patient presented significant clinical improvement after initiation of corticosteroid treatment.

**Figure 1:**
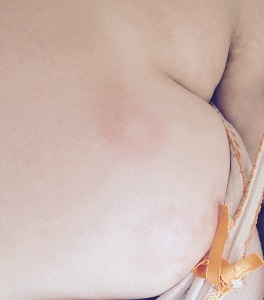
A salmon-colored rash, appearing with the fever in the evening hours and vanishing during apyrexia, located at patient's breast

